# Supplementary material for: Quorum sensing mediates morphology and motility transitions in the model archaeon Haloferax volcanii
Source: mBio. 2025 Jun 18;16(7):e00906-25. doi: 10.1128/mbio.00906-25 (PMC12240190; doi:10.1128/mbio.00906-25)
Supplement: Volcano plots — Interactive volcano plots. [file mbio.00906-25-s0002.html]

Supplementary Material


# Supplementary Material

#### Priyanka Chatterjee et. al.

#### 2024-11-05

Below are interactive volcano plots of the quantitative proteomics
data from Figure 4. See Fig. 4 legend for more information.

## Wild type at early-log vs. wild type at late-log

## Wild type at early-log, regular medium vs. 1% conditioned medium

## Deletion strain of DdfA at early-log, regular medium vs. 1% conditioned medium
